# Effect of Piezoelectric BaTiO_3_ Filler on Mechanical and Magnetoelectric Properties of Zn_0.25_Co_0.75_Fe_2_O_4_/PVDF-TrFE Composites

**DOI:** 10.3390/polym14224807

**Published:** 2022-11-08

**Authors:** Kirill Sobolev, Valeria Kolesnikova, Alexander Omelyanchik, Yulia Alekhina, Valentina Antipova, Liudmila Makarova, Davide Peddis, Yuriy L. Raikher, Katerina Levada, Abdulkarim Amirov, Valeria Rodionova

**Affiliations:** 1 Research and Education Center “Smart Materials and Biomedical Applications”, Immanuel Kant Baltic Federal University, 236014 Kaliningrad, Russia; 2 Faculty of Physics, Lomonosov Moscow State University, 119991 Moscow, Russia; 3 Department of Chemistry, University of Genova, 16146 Genova, Italy; 4 Institute of Natural Sciences and Mathematics, Ural Federal University, 620000 Ekaterinburg, Russia

**Keywords:** polymer composites, multiferroics, PVDF-TrFE, magnetoelectric effect, scaffold-aided bone repair

## Abstract

Polymer-based multiferroics, combining magnetic and piezoelectric properties, are studied experimentally—from synthesis to multi-parameter characterization—in view of their prospects for fabricating biocompatible scaffolds. The main advantage of these systems is facile generation of mechanical deformations and electric signals in response to external magnetic fields. Herein, we address the composites based on PVDF-TrFE polymer matrices filled with a combination of piezoelectric (BaTiO_3,_ BTO) and/or ferrimagnetic (Zn_0.25_Co_0.75_Fe_2_O_4_, ZCFO) particles. It is shown that the presence of BTO micron-size particles favors stripe-type structuring of the ZCFO filler and enhances the magnetoelectric response of the sample up to 18.6 mV/(cm∙Oe). Besides that, the admixing of BTO particles is crucial because the mechanical properties of the composite filled with only ZCFO is much less efficient in transforming magnetic excitations into the mechanical and electric responses. Attention is focused on the local surfacial mechanical properties since those, to a great extent, determine the fate of stem cells cultivated on these surfaces. The nano-indentation tests are accomplished with the aid of scanning probe microscopy technique. With their proven suitable mechanical properties, a high level of magnetoelectric conversion and also biocompatibility, the composites of the considered type are enticing as the materials for multiferroic-based polymer scaffolds.

## 1. Introduction

The growing number of pathologies of the musculoskeletal system requires the search for new effective methods of bone tissue restoration [1]. Tissue engineering and regenerative medicine offer new strategies for bone tissue repair using scaffolds that stimulate stem cell differentiation towards the osteogenic lineage [2,3]. To reproduce the optimal conditions for differentiation of stem cells in vitro/in vivo on scaffolds, it is necessary to take into account the structural features of bones (stiffness, porosity, internal nanostructure), and also to ensure the action of various biophysical signals, such as piezoelectric, pyroelectric, ferroelectric, etc., since they play a key role in the regulation of the stem cells’ activity [3,4,5].

Biophysical stimulation, exerted under various environmental conditions like mechanical stiffness, adhesion ability and nanoscale organization of the scaffold [4,6], can drive stem cell differentiation in the neuronal [7,8,9], myogenic [10] and osteogenic [11,12] directions. To a large extent, this choice depends on the properties of the scaffold base material. The variety of those used for bone tissue engineering includes metal oxides (e.g., TiO_2_), bioceramics (BaTiO_3_, MgSiO_3_, (Na,K)NbO_3_-based ceramics, etc.), natural (collagen, keratin, fibrin, cellulose, chitosan) and synthetic polymers (e.g., polyvinylidene fluoride (PVDF) or poly(vinylidene fluoride–trifluoroethylene) (PVDF-TrFE)) [5,13,14].

Natural polymers with piezoelectric properties are very much appropriate as scaffold bases [15,16]. However, their high degradation rate and low stiffness significantly reduce the mechanical endurance of the resulting scaffolds. To improve the functionality, attempts have been undertaken [5,17,18] to introduce natural piezoelectric polymers, collagen in particular, into the scaffolds, based on synthetic polymers. Another prospective way to obtain scaffolds with an optimal set of piezoelectric and mechanical properties is to combine piezoceramics and piezopolymers into a composite material [19].

In such materials, the electrical polarization mediating the cell activity can be remotely controlled by ultrasound [9]. However, acoustic excitation is not the only conceivable means to accomplish that. An alternative way to induce the intrinsic piezopolarization in the scaffolds is the magnetoelectric effect, provided the scaffold is made of an appropriate multiferroic substance. Remarkably, in that case, one is relieved by definition of all the problems of finding a technologically optimal choice for the transducer position, matching of wave resistances between the emitting device and a target, occurrence of parasite reflections of the beam, etc. Besides that, the magnetoelectric effect can truly be induced remotely (no direct contact with the field source is needed). All the tissues of a human’s body are ‘transparent’ to a magnetic field whose set of parameters—direction, amplitude and frequency—may be varied in wide ranges. Development of such multiferroic materials, where the piezoelectric and magnetic properties are inherently coupled, has received a great deal of attention in recent years [20,21,22,23,24].

Multiferroic technology responds to the general demand for miniature, lightweight, flexible elements made of easily processed materials. The magnetoelectric conversion in such structures occurs due to the mechanical stresses, generated by magnetostriction or magnetostatic interactions of the filler, which induce electric polarization of the piezopolymer matrix. Such composites not only overcome limitations associated with the rigidity and fragility of the classical ceramic-based or laminar materials, but are also leak-resistant and in some cases biocompatible [24].

PVDF and its copolymers, PVDF-TrFE in particular, are the most common piezopolymers for creating composite structures. PVDF has several different polymorphs, among which the polarized β-phase is the best in terms of piezoelectric properties [25,26]. Previously it was reported that the addition of a ceramic filler leads to an increase in the material’s dielectric constant; the interaction of the polar molecules of PVDF or copolymer with the charged surface of the inclusion particles causes polymer ordering, nucleation of the electroactive β-phase and the appearance of an additional charge [27,28,29,30,31]. Due to the interface nature of this effect, phase formation in piezopolymer depends on the size and volume fraction of the filler. The largest fraction of the electroactive phase is formed at low concentrations (15 wt.%) of the filler [28,29]. Polymerization of PVDF with other polymers, PVDF-TrFE in particular, by itself also increases the piezoelectric response of the material, enhancing formation of the β-phase due to appearance of extra fluorine molecules in its composition and the associated steric effect [32].

There are various methods for PVDF/PVDF-TrFE processing and scaffold fabrication, such as solvent casting, electrospinning, doctor blade, fused deposition, 3D printing and selective laser sintering, etc. [33,34]. Modification of the standard protocols for piezopolymers processing makes it possible to create scaffolds with improved piezoelectric properties, thereby efficiently modulating the cellular activity. For example, more than once it has been reported that inclusion of ferroelectric BaTiO_3_ particles in PVDF-TrFE-based composites improves their piezoelectric properties, thus facilitating osteogenic differentiation and bone growth [35,36].

Notably, when designing an optimal scaffold-oriented PVDF-based multiferroic, one has to comply with a number of restrictions. For example, a large amount of a piezoelectric filler leads to the loss of flexibility in the composite [5,37]. Moreover, enriching the ceramic content of the composite may cause deterioration of its mechanical and piezoelectric characteristics [28,29]. Meanwhile, a wide-range tuning of the stiffness of the scaffold is vitally important for the development of stem cells inhabiting its surface. Indeed, whereas neuronal differentiation is observed on soft substrates (≤10^3^ Pa), for osteogenic differentiation one needs sufficiently hard substrates (≥10^6^ Pa). Within these limits, i.e., on the scaffolds with stiffnesses between 10^3^ to 10^6^ Pa, stem cells may differentiate in the neuronal, myo- or osteogenic directions, depending on the cultivation conditions and the type of cells [4]. Notably, the cultivated cells respond to the mechanical properties of the surfacial area of the scaffold, not to those arising from the bulk. Thus, it is essential to employ local, nanoscale methods to probe the mechanical properties, for example, nano-indentation by means of scanning probe microscopy (SPM), which renders realistic information on the surfacial mechanical response [38,39,40]. Therefore, the general goal is to work out the methods for producing advanced composites which possess both a high piezoelectric response and appropriate mechanical properties.

Earlier, in ref. [41], we studied nanocomposites based on PVDF and PVDF-TrFE filled with magnetic nanoparticles made of two types of spinel ferrites. Two ways of increasing the magnetoelectric response were attempted: (*i*) polymerization under an external magnetic field in order to align magnetic particles, and (*ii*) adding a third component: ferroelectric BaTiO_3_ particles. Nanocomposites of CoFe_2_O_4_ (CFO) nanoparticles in PVDF-TrFE matrix, aligned by the magnetic field, showed the highest value of the magnetoelectric coefficient: $\alpha_{\mathrm{ME}}\sim$18.5 mV/(cm∙Oe). In this work, the same methodology is applied to produce the aligned composites based on PVDF-TrFE and Zn-doped cobalt ferrite Zn_0.25_Co_0.75_Fe_2_O_4_ (ZCFO) magnetic nanoparticles, both with and without inclusion of piezoelectric BaTiO_3_ particles.

ZCFO was chosen due to its enhanced saturation magnetization $M_{s}$ and augmented magnetic softness, i.e., lower anisotropy. Larger values of $M_{s}$ (74 emu/g for ZCFO vs. 66 emu/g for CFO) provide an almost 25% increase in the force the particles exert on the matrix under the external magnetic field, whereas the improved magnetic softness ensures a better return of the magnetized composite to its initial state after the external field is off. Moreover, in this article we investigate for the first time the effect of the filler (magnetic, ferroelectric or mixed) on the surficial mechanical properties of nanocomposites in comparison with pure PVDF-TrFE film. As mentioned, understanding the mechanical response of composites with different types of fillers in connection with their magnetoelectric performance is necessary for advancing the design of materials for biomedical scaffolds.

## 2. Materials and Methods

### 2.1. Synthesis and Properties of the Filler Particles

Synthesis and detailed morphological and structural characterization of Zn_0.25_Co_0.75_Fe_2_O_4_ (ZCFO) nanoparticles are given in refs. [41,42]. The same nanoparticles for this work were prepared by the sol-gel self-combustion method [43]. The crystallite size estimated from XRD was 16 ± 2 nm. The magnetization curve was measured with a Lake Shore 7400 (Lake Shore Cryotronics, Inc., Westerville, OH, USA) vibrating sample magnetometer (VSM) on a dry powder at 297 K. The M(H) curve is loop-shaped with saturation magnetization of 74 ± 2 emu/g, *M_R_/M_S_* of 0.34 and coercivity of 540 ± 20 Oe [41,42]; here, *M_R_* stands for remanence.

BaTiO_3_ (BTO) particles were prepared by the solid-state reaction method followed by a two-stage sintering: at *T_1_* = 1150 °C during τ_1_ = 4 h (1st stage) and at *T_2_* = 1170 °C during *τ_2_* = 4 h (2nd stage). For this procedure, BaCO_3_ and TiO_2_ powders with purity of at least 99.95% were used as precursors. The fabrication method is in line with the conventional ceramic technology, described in detail elsewhere [44]. The resulting powder has reference size of 3 ± 1 µm. After synthesis the particles were polarized in the DC electric field *E* = 20 kV/cm.

### 2.2. Fabrication of Magnetoelectric Nanocomposites (NCs)

**Figure 1:**
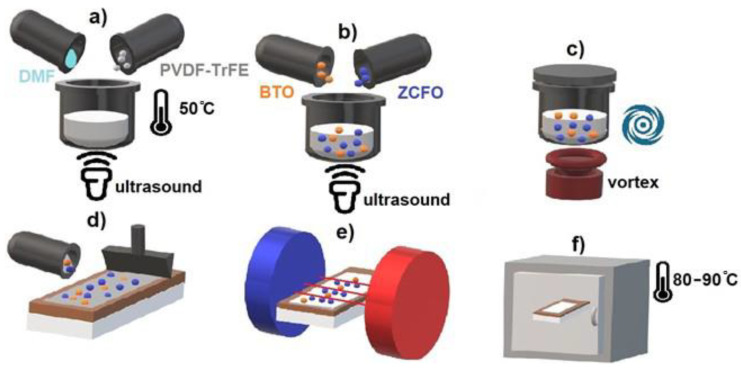
Scheme of the nanocomposite fabrication process. (**a**) Preparation of the base solution, (**b**) Adding fillers, (**c**) Blending, (**d**) Spreading, (**e**) Alignment in a magnetic field, (**f**) Evaporation.

Nanocomposites were fabricated by the solvent casting method, assisted by the doctor blade technique [33]. For all the samples the protocol of [33] was modified: solvent evaporation was performed in presence of the external magnetic field of 1 kOe, applied in the film. The protocol consisted of the following steps and is schematically illustrated in Figure 1.

1. Preparation of the base solution (Figure 1a).

For this purpose, the powder of poly(vinylidene fluoride) copolymer with trifluoroethylene PVDF-TrFE 55/45 (Piezotech, King of Prussia, PA, USA) was dissolved in dimethylformamide (DMF), used as a solvent, in a weight ratio of 1:12. Complete dissolution of PVDF-TrFE and obtaining of a homogeneous solution was achieved by using an ultrasonic bath for 45–60 min at a heating temperature of about 50 °C.

2. Adding fillers (Figure 1b).

At this stage, ferromagnetic ZCFO and/or ferroelectric BTO particles were added to the prepared PVDF-TrFE base solution and mixed for at least 60 min using the ultrasonic bath.

3. Blending (Figure 1c).

The obtained PVDF-TrFE solution with ferromagnetic ZCFO and/or ferroelectric BTO fillers was re-mixed before the final spreading using a laboratory mixer (Vortex type).

4. Spreading (Figure 1d).

The final solution was spread on a glass substrate and leveled with a special blade to obtain the uniform layer.

5. Alignment in a magnetic field (Figure 1e).

The external magnetic field of about 1 kOe was applied parallel to the plane of the substrate with the final solution.

6. Evaporation (Figure 1f).

At the final stage, the samples were obtained by the evaporation of the solvent, for which the solution, prepared on the glass substrates, was placed in an oven and dried at the constant temperature of 85 °C for 45 min.

Four types of films with thickness of 100–150 µm were produced under the same preparation conditions. Hereafter, we denote them as follows:

- PVDF-TrFE—pure PVDF-TrFE polymer;
- BTO/PVDF-TrFE—a composite filled with 5 wt.%. of BTO particles;
- ZCFO/PVDF-TrFE—a composite filled with 15 wt.% of ZCFO particles;
- BTO/ZCFO/PVDF-TrFE—a composite filled with 5 wt.% of BTO and 15 wt.% of ZCFO particles.

For use in the measurements, from each film a sample with dimensions of 10 × 5 mm was cut with the longer side along the direction of the applied magnetic field.

### 2.3. Structural Characterization (X-ray Computer Tomography, XRD, SEM-EDX)

High resolution X-ray computed tomography (XCT) was used to obtain 3D mapping of the X-ray optical density in the bulk of ZCFO/PVDF-TrFE and BTO/ZCFO/PVDF-TrFE samples. The particle distribution was visualized with the aid of XCT YXLON (Cheetah, Germany) that ensures spatial resolution not less than 1 µm.

The surface microstructure of the samples was analyzed with TM4000II Tabletop Scanning Electron Microscope (Hitachi Ltd., Tokyo, Japan). In each case, we used backscattered electrons (BSE) contrast, sensitive to the chemical composition. The spatial distribution of chemical elements was analyzed with a QUANTAX 75 EDX Detector (Bruker Nano GmbH, Berlin, Germany) for three levels of the accelerating voltage, namely *U_a_* = 5, 10 and 15 keV, corresponding to the effective depths of signal acquisition 0.4, 1.3 and 2.9 µm, respectively. The obtained data enables one to estimate the mean depth of the ferrite particles (which are cytotoxic) bedding below the surface of the polymer which itself is biocompatible.

### 2.4. Magnetic Characterization (VSM, FORC)

VSM, mentioned in Section 2.1, was used to investigate the magnetic properties of ZCFO-containing samples in the field up to 10 kOe at room temperature (~297 K). The measuring field H was directed: (*i*) in-plane under angles 0°, 45° and 90° to the direction of the sample texture formed during polymerization, and (*ii*) out-of-plane, either normally to the film or under 45° to the film normal and 90° to the structure direction.

Standard measurements of *M(H)* curves do not reveal any significant differences between ZCFO-containing samples. As a more sensitive test, FORC analysis (First Order Reversal Curve) [45,46,47,48] was used with the aid of 7400 VSM FORC Utility and DoFORC software of Cimpoesu et al. (2019) [45]. This experimental method provides information about the switching and interaction fields of all the magnetic phases present in the sample [47,48,49]. Moreover, in composite materials such as elastomers, FORC analysis is useful for detecting the effect of the polymer stiffness on the magnetization processes [49,50,51,52].

### 2.5. Atomic and Magnetic Force Microscopy (AFM, MFM) and Magnetoelectric Properties

Mechanical properties of the samples were examined by scanning probe microscopy (SPM). We used NTEGRA SPM (NT-MDT, Zelenograd, Russia) in the nano-indentation mode [53] when at each point of the scan the force-versus-distance curves are being recorded. By analyzing these curves both on collision and retraction, the morphology, mechanical stiffness of the surface and its Young’s modulus were evaluated. We performed simultaneous mapping of all these properties in several locations on the surface of each sample to get average reference values. The NSG10 tips with 3 nm curvature radius were used; all the scans had 500 × 500 points dimensionality.

**Figure 2:**
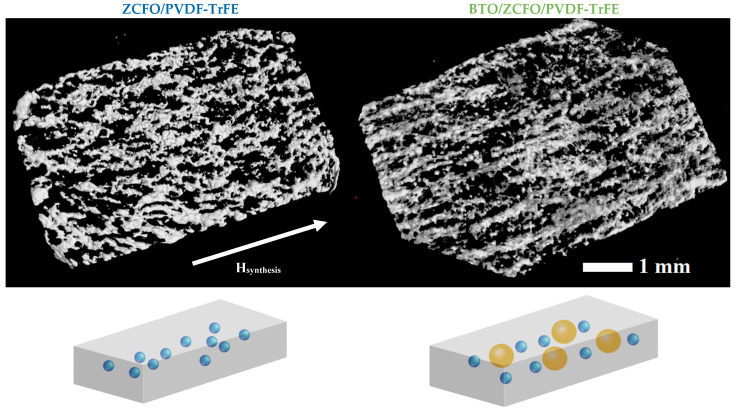
Post-processed 3D XCT scans of ZCFO/PVDF-TrFE (**left**) and BTO/ZCFO/PVDF-TrFE (**right**) samples, demonstrating the filler subsystem without the polymer matrix.

As seen in Figure 2 (below), ZCFO nanoparticles, having been admixed to the PVDF-TrFE matrix, agglomerate during polymerization. To elucidate the nature and strength of interaction between the matrix and filler clusters, we have done the following. Multiparticle ZCFO aggregates present inside the film were located by measuring the Magnetic Force Microscopy (MFM) contrast. For that, ZCFO/PVDF-TrFE and BTO/ZCFO/PVDF-TrFE samples were scanned with Co-covered MFM01 tips of 8 nm curvature radius. The scans of 500 × 500 points dimensionality were obtained by the standard two-path approach: 100 nm tip lift on the second path. Then, the stray field distribution was registered under a sequence of external fields (0, 50, 100, 200 and 400 Oe), exerted in the scanning plane perpendicular to the direction of the field, applied during the solvent evaporation. For each field value, a quick 256 × 256 points MFM-scan was performed without a tip retraction from the surface. This was done to reveal and visualize the motion of ZCFO agglomerates inside the matrix in order to correlate those changes with the observed mechanical, magnetic and magnetoelectric properties.

The dependences of the magnetoelectric coefficient on temperature, magnetic field and the inclination angle of the latter, were obtained for ZCFO/PVDF-TrFE and BTO/ZCFO/PVDF-TrFE samples using the method discussed elsewhere [41]. Briefly, we employed a custom-designed setup with a lock-in amplifier (Model SR830, Stanford Research, Sunnyvale, CA, USA). This setup ensures measuring of the magnetoelectric response with precision higher than 1% under external fields up to 10 kOe oriented within 0–180° to the sample surface; the accessible temperature range is from room temperature to about 360 K.

## 3. Results and Discussion

### 3.1. Structural Characterization

Front-view 3D images of ZCFO/PVDF-TrFE and BTO/ZCFO/PVDF-TrFE films, plotted on the base of the XCT layer-by-layer scanning data together with schematic representations of the observed structures, are shown in Figure 2. Data points corresponding to homogeneous polymer regions were removed from the images to improve the view of filler particles. In both samples, the stripe-like agglomerates of ZCFO nanoparticles are present which had been formed as a result of the magnetic field application during synthesis. Notably, in BTO/ZCFO/PVDF-TrFE the orientation order in the stripe-like structure is more pronounced.

One of the possible explanations for the enhanced agglomeration of ZCFO nanoparticles in BTO/ZCFO/PVDF-TrFE is the augmented crystallization of the matrix provoked by the BTO particles. Note that, as the XCT data show, spontaneous ZCFO cluster formation takes place in the samples with a relatively high (>10 wt.%) filler content. All the samples were prepared with 15% weight content of ZCFO MNPs since, according to the literature, when maintaining this concentration of MNPs, the highest value of magnetoelectric coefficient is achieved [41]. Thus, ZCFO-subsystem is prone to agglomeration even without BTO. Meanwhile, BTO particles nucleate additional spherulites around them which, when growing, expel ZCFO to their outer boundaries. The augmented confinement of ZCFO nanoparticles favors their grouping in dense stripe-like structures whose alignment is imposed by the external field. We infer that the field-induced structuring in the BTO/ZCFO/PVDF-TrFE sample plays a key role in the formation of its mechanical properties which, in turn, should strongly affect the bioactive performance of the composites.

**Figure 3:**
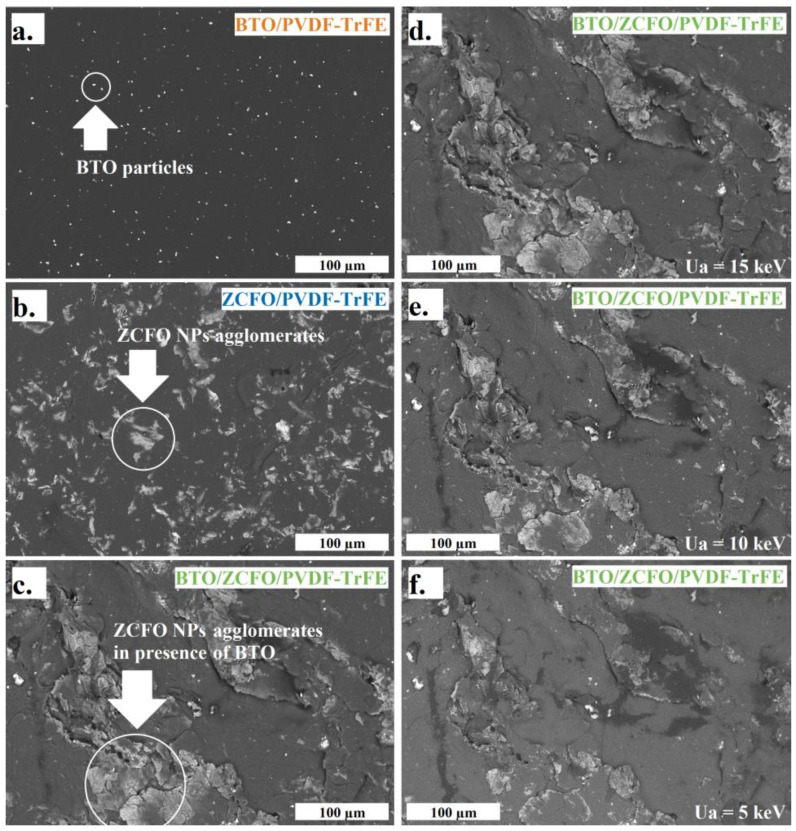
SEM (BSE) micrographs of BTO/PVDF-TrFE (**a**), ZCFO/PVDF-TrFE (**b**) and BTO/ZCFO/PVDF-TrFE (**c**,**d**) samples under *U_a_* = 15 keV; the same area of BTO/ZCFO/PVDF-TrFE sample under *U_a_* = 10 keV (**e**) and *U_a_* = 5 keV (**f**); the sale bar is the same for all the panes.

SEM-images of all the tested samples, obtained with *U_a_* = 15 keV (maximal resolved depth), are shown in Figure 3a–d. Figure 3a evidences fairly homogeneous distribution of BTO microparticles that agrees well with the XCT results. In the ZCFO/PVDF-TrFE sample (Figure 3b), the ZCFO-filler-rich regions (‘islands’) are visible across the whole area with no evident singled-out orientation. Meanwhile, in the image corresponding to the BTO/ZCFO/PVDF-TrFE sample (Figure 3c), ZCFO particles form a stripe region surrounded by almost clean matrix. This structure difference is in full agreement with the obtained XCT data.

The images in Figure 3d–f present the SEM data on the same region of the BTO/ZCFO/PVDF-TrFE sample taken under variations of *U_a_*. Quite visibly, the scans become more blurred with the decrease of *U_a_*. This evidences that the particles are mostly located inside the bulk of the film. This conclusion is supported by the data on distribution of chemical content of the film over its depth presented in Table 1. An example of corresponding *U_a_*-dependent mapping for ZCFO/PVDF-TrFE sample is given in Figure S1. We note that in none of the samples did EDX analysis reveal the filler particles (either ZCFO or BTO) closely abutting on the surface. Indeed, the peaks in the EDX spectra, inherent to metals, appear only at higher accelerating voltages. This confirms—in agreement with the results obtained on similar samples [41]—that all the metal-containing particles are embedded in the polymer bulk, thus ensuring that the surfaces of the studied films remain biocompatible. Also, oxide filler particles induce oxygen to the EDX spectra, which was not there for the pure PVDF-TrFE matrix sample.

### 3.2. Magnetic Characterization

Macroscopic magnetic properties of the composite samples were studied with VSM at room temperature. Figure S2 shows the angular dependences of M(H) loops recorded on ZCFO/PVDF-TrFE and BTO/ZCFO/PVDF-TrFE samples for the in-plane and out-of-plane orientations of the applied field. The obtained respective values of coercivity fields do not differ much being about 500 Oe. The in-plane hysteresis loops (Figure S2a,c) are isotropic and very similar to those reported in ref. [41]. The out-of-plane loops (Figure S2b,d) exhibit a small difference that may be attributed to the shape anisotropy of the film which is weak due to a low value of the sample magnetization. This establishes that the effect of incorporation of BTO microparticles in ZCFO/PVDF-TrFE films on the macroscopic magnetic properties of the composite is not strong.

**Figure 4:**
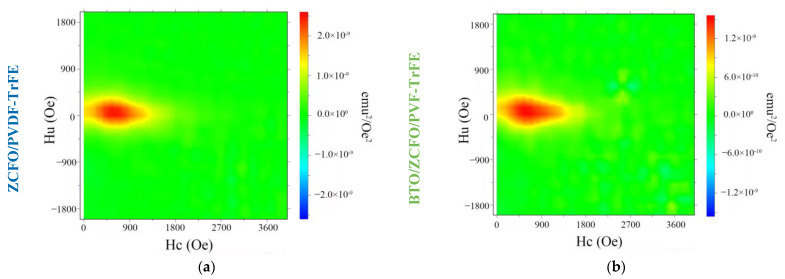
FORC diagrams of ZCFO/PVDF-TrFE (**a**) and BTO/ZCFO/PVDF-TrFE (**b**) composite samples.

Despite being poorly distinguishable for plain *M(H)* loops, this effect is resolved with the aid of FORC analysis. In Figure 4, the FORC diagrams of the abovementioned samples are presented. They are plotted in the conventional (*H_c_, H_u_*) plane where *H_c_* is a coercive field and *H_u_* is a bias (interaction) field [44,52,54,55]. For each of the samples under study, the distribution of those parameters is rendered by an ellipse-like patch at the FORC-plane with dimensions *ΔH_c_* and *ΔH_c_*, which are the lengths of the major and minor axes of the patch, respectively. For the ZCFO/PVDF-TrFE sample one finds *ΔH_c_*^(1)^ = 1100 Oe and *ΔH_u_^(^*^1)^ = 500 Oe, whereas for the BTO/ZCFO/PVDF-TrFE sample *ΔH_c_^(^*^2)^ = 1200 Oe and *ΔH_u_*^(2)^ = 550 Oe. Moreover, further growth of *ΔH_u_*^(2)^ is expected with the increase of local concentration of the magnetic fraction [56].

The distribution of coercive fields in both cases reflects the distribution of switching fields between particles and clusters of different sizes [47]. In the sample with BTO particles, the FORC diagram shifts rightward along the *H_c_* axis, which could be attributed to the influence of ferroelectric particles on the structural properties of the polymer and the possibility of displacement of ferromagnetic particles inside the matrix [57,58,59].

### 3.3. Mechanical, Surface and Magnetoelectric Properties

Examples of mapping surface morphology, mechanical stiffness, Young’s modulus and adhesion force are shown in Figure S3. The averaged values of the mechanical stiffness and Young’s modulus for the studied composite films, together with the respective magnetoelectric coefficients, are summarized in Table 2. As can be seen, the ZCFO/PVDF-TrFE sample (only ferrite filler) differs from all the others by being considerably weaker with respect to all the mechanical characteristics. Meanwhile, adding a small amount of BTO brings the mechanical properties back to the values inherent to pure matrix. Most probably, when a notable amount of nanosize solid phase is embedded, the polymer encapsulates the filler clusters and a greater fraction of it remains non-crystallized [28,29]. As a result, the sample becomes less stiff. As was previously described, the ceramic additives act as nucleation agents for the polymer spherulites. However, for large filler concentrations, a lower level of crystallinity is reported due to the reduced chain mobility and the hindrance of crystal growth due to the neighboring spherulites [28]. As the polymer encapsulates the filler particles, a weakening of the polymer connectivity might appear, leading to sliding of the encapsulated clusters. This effect is observed as the lowering of Young’s modulus, which was shown, for example, in ref. [60].

In the case of mixed filler, BTO microparticles serve as crystallization-aiding substrates, supporting formation of ferrite-free spherulites. This, on the one hand, reduces the size of ferrite-rich regions, which in turn makes the stripe structure more pronounced; cf. the images in Figure 2. On the other hand, in the ferrite-free regions, crystallization goes unobstructed, so that the mechanical properties approach those of the pure or just BTO-admixed PVDF-TrFE, in accordance with the evidence reported in [30,41]. Therefore, in the mechanical aspect, BTO/ZCFO/PVDF-TrFE composites, according to [4], are quite appropriate for making scaffolds aimed at osteogenic stem cells’ differentiation.

**Figure 5:**
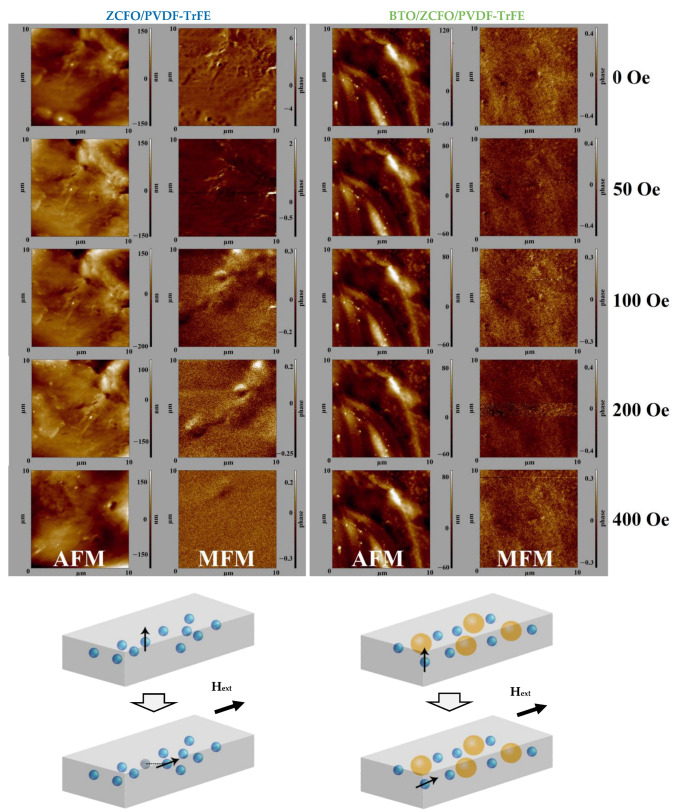
AFM-MFM images of ZCFO/PVDF-TrFE and BTO/ZCFO/PVDF-TrFE samples under application of magnetic field *H* = 0, 50, 100, 200 and 400 Oe.

AFM and MFM images, taken on the same parts of the surface of ZCFO/PVDF-TrFE and BTO/ZCFO/PVDF-TrFE samples under the field varied from 0 to 400 Oe, are given in Figure 5. Initially (upper row in Figure 5), the magnetic contrast maps of both samples reflect the distribution of the stray fields of ZCFO agglomerates. Indeed, the size distribution of ZCFO aggregates is rather wide, from tens to many hundreds of nanometers. Because of that, the orienting action of the magnetic field during synthesis by no means can ensure a perfect in-plane magnetic structure. The scans’ evidence, however, for the extent of uniformity of magnetization field-induced during solidification is considerable; the number of strong MFM contrast loci is few in both samples.

Application of the probing field of moderate strength (below 400 Oe) reveals an essential difference between the samples with respect to the mobility of ZCFO aggregates and, thus, to the rheology of the respective composites (see the schemes at the bottom of Figure 5). Whereas the MFM map of the ZCFO/PVDF-TrFE sample changes drastically with the probing field increase, the view of BTO/ZCFO/PVDF-TrFE hardly changes at all.

The similarity of scans recovered under the enhanced probing field (400 Oe) is due to the fact that the magnetization finally complies with the direction of the latter. This re-entering of the uniformity state is expectable given a relative freedom of the aggregates in ZCFO/PVDF-TrFE films. At the same time, we barely observe any changes on the AFM scans for the BTO/ZCFO/PVDF-TrFE sample under the external magnetic field, whereas in the ZCFO/PVDF-TrFE sample the changes are well pronounced, causing variation of the surface morphology. This is also due to the enhanced mobility of ZCFO agglomerates. All this evidence is entirely coherent with the results of the direct mechanical tests given in Table 2.

Moreover, the MFM scanning tests, where the probing field is switched on/off, indicate a pronounced viscoelasticity. According to our observations, unlike the BTO/ZCFO/PVDF-TrFE sample, the ZCFO/PVDF-TrFE one never recovers the initial state in full. This is an additional proof of the poor crystallization of PVDF-TrFE matrices with only ferrite nanoparticles embedded.

**Figure 6:**
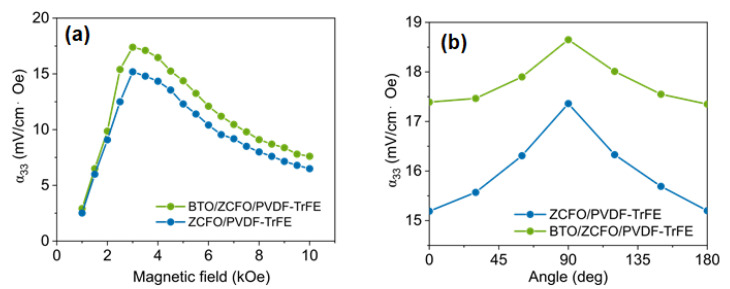
Dependences of ME voltage coefficients (α_ME_) of ZCFO/PVDF-TrFE and BTO/ZCFO/PVDF-TrFE samples on: (**a**) the amplitude $H_{\mathrm{AC}}$ of the applied magnetic field at room temperature; and (**b**) the angle between the field direction and the normal to the film plane (room temperature).

The dependences of the ME voltage coefficient (α_ME_) on the amplitude *H_AC_* of external AC magnetic field with frequency of 10 kHz, and on the field tilt to the film plane, are shown in Figure 6. The field-amplitude dependences of both the ZCFO/PVDF-TrFE and BTO/ZCFO/PVDF-TrFE films display a peak-like behavior with a maximum at ~3 kOe (see Figure 6a), which is about thrice stronger than the field imposed during synthesis. Earlier [41], the same tendency—the increase of the ME effect upon adding a small amount of ferroelectric micron-size particles—was reported for composites based on CoFe_2_O_4_ nanoparticles. This enhancement can be attributed to different factors, e.g., additional piezoelectric effects of BTO particles, or higher formation of the electroactive β-phase in the matrix in the presence of BTO particles. In our case, the relation between the maximal values of α_ME_ is the same (see Table 2 and Figure 6a): 17.4 and 18.6 mV/(cm∙Oe) for ZCFO/PVDF-TrFE and BTO/ZCFO/PVDF-TrFE, respectively.

Occurrences of that difference in α_ME_ may be justly attributed to the difference in mechanical properties between the BTO- and non-BTO ZCFO/PVDF-TrFE films. Figure 6b shows that for ZCFO/PVDF-TrFE the effect is less pronounced but more sensitive to the field direction. Both these features are well understandable given the enhanced positional and orientation freedom allotted to the ZCFO particles by a polymer matrix with a lower level of crystallization.

**Figure 7:**
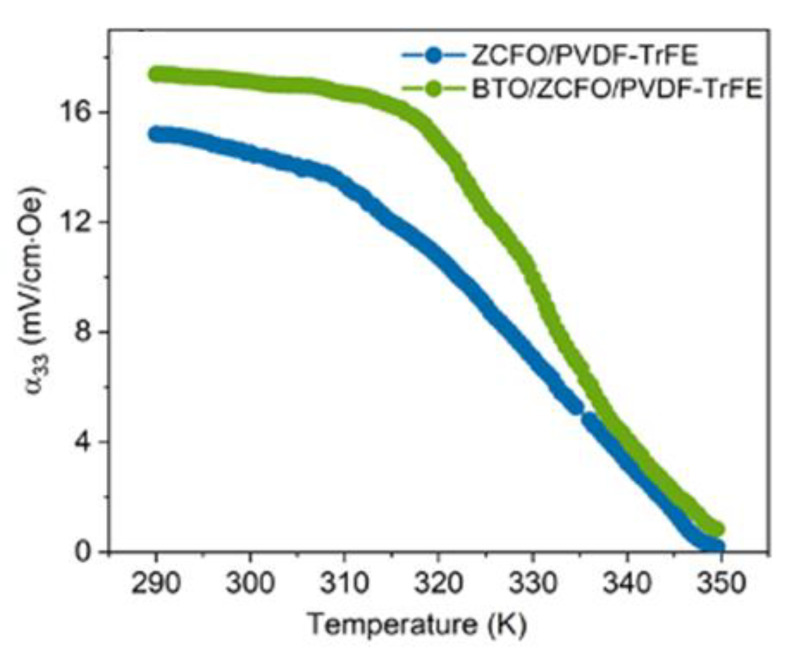
Temperature dependences of ME voltage coefficients ($\alpha_{\mathrm{ME}}$) of ZCFO/PVDF-TrFE and BTO/ZCFO/PVDF-TrFE samples for $H_{\mathrm{AC}}=$ 3 kOe and f= 10 kHz.

As is seen from Figure 7, both the ZCFO/PVDF-TrFE and BTO/ZCFO/PVDF-TrFE composites demonstrate diminution of the ME coefficient to virtually zero within an interval from about 315 K to 350 K. This type of behavior is clearly imposed by the PVDF-TrFE matrix which at about 350 K undergoes the ferroelectric–paraelectric phase transition [61]. In other words, the presence of solid admixtures does not affect significantly the temperature behavior of the magnetoelectric response of the composites. However, the higher stability of the BTO/ZCFO/PVDF-TrFE structure makes this material a bit more heat-tolerant.

It is instructive to compare the obtained values of α_ME_ with those of similar PVDF or PVDF-TrFE based composites. P. Martins et al. [62] measured the ME coefficient of X^2+^Fe^3+^_2_O_4_/PVDF-TrFE (X^2+^ = Zn/Mn, Co and Fe) fabricated using hydrothermal method. The reported highest value of the ME coefficient for the systems with CoFe_2_O_4_ nanofiller measured under the field 2600 Oe was 6.5 mV/(cm∙Oe). Therefore, the here-reported ME coefficients are more than twice as high.

In connection with the biological aspect of the problem, we note the results in ref. [63]. There, proliferation of preosteoblast cells MC3T3-E1 was succesfully established by magnetomechanical and ME stimulation on the scaffolds made of CFO/PVDF composites with mechanical properties much weaker and magnetoelectric response much lower than those demostrated by our BTO/ZCFO/PVDF-TrFE systems. On that basis, we infer that the here-presented composites are indeed very appropriate materials for scaffold-mediated bone tissue repair.

## 4. Conclusions

By means of numerous experimental techniques, it is demonstrated that combined embedding of magnetic and ferroelectric (ZCFO and BTO) nano- and micron-sized particles into PVDF-TrFE polymer matrices yields a composite whose set of mechanical and electroactive properties nicely fits the requirements for a material aimed at scaffold-mediated repairs of bone tissue. The role of each component is demostrated by investigating the auxiliary systems, each of which lacks both or one of the solid components, namely, pure PVDF-TrFE, BTO/PVDF-TrFE and ZCFO/PVDF-TrFE films that are run through the same number of tests. This comparative analysis has shown that only the synergy imparted by interaction of both BTO and ZCFO with the matrix provides the desired set of properties. The latter comprises a high value for the magnetoelectric coefficient (18.6 mV/(cm∙Oe)) and sufficient stiffness (0.028 N/mm). Moreover, the viscoelasticity (retardation effect) of the BTO/ZCFO/PVDF-TrFE composite is low, which ensures reproducibility of its mechanical and electric responses under repetitive stimulations. The main stimulating agent is external magnetic field that, acting directly on the spinel ferrite (ZCFO) phase, by changing the interparticle magnetostatic forces induces both direct mechanical stresses acting on the matrix and, implicitly, via stressing the piezoelectric particles, causes them to electrically polarize. Given that, the scaffolds made of the aforementioned composite should be able to efficiently change the biophysical parameters of stem cells’ microenvironment and modulate cell activity. Further research will be aimed at improving the adhesive properties of the composites and introducing surface functionalization, as well as studying the effect of the external magnetic field on the surface properties.

## Figures and Tables

**Table 1:** Elemental atomic content (C, O, F, Fe and Co) at different depths (2.9, 1.3 and 0.4 µm) from acquired EDX signal. The evaluation errors are: 1–2% for C, F and O, and 0.1–0.3% for all other elements.

Depth, µm	Atomic Content, %				
	C	O	F	Fe	Co
	PVDF-TrFE				
2.9	41	-	59	-	-
1.3	40	-	60	-	-
0.4	36	-	64	-	-
	BTO/PVDF-TrFE				
2.9	45	1.2	54	-	-
1.3	46	1.4	53	-	-
	ZCFO/PVDF-TrFE				
2.9	45	2.4	52	1.0	0.4
1.3	45	2.6	52	0.6	-
0.4	44	3.1	52	-	-
	BTO/ZCFO/PVDF-TrFE				
2.9	52	6.8	38	1.5	0.6
1.3	54	7.3	37	1.1	-
0.4	56	10.6	31	-	-

**Table 2:** Concentration, mechanical and magnetoelectric parameters for the studied samples.

Sample	Filler Content, wt.%	Young’s Modulus, MPa	Mechanical Stiffness, mN/μm	$\mathrm{Max}\;\mathrm{of}\;\alpha_{ME},\;\mathrm{mV}/(\mathrm{cm}\cdot \mathrm{Oe})$
PVDF-TrFE	-	0.63	0.027	-
BTO/PVDF-TrFE	5	0.65	0.028	-
ZCFO/PVDF-TrFE	15	0.3	0.008	17.4
BTO/ZCFO/PVDF-TrFE	5 (BTO) 15 (ZCFO)	0.65	0.028	18.6

## Data Availability

Not applicable.

## Supplementary Materials

The following supporting information can be downloaded at: https://www.mdpi.com/article/10.3390/polym14224807/s1, Figure S1: Representative EDX mapping, obtained for ZCFO/PVDF-TrFE sample, with different *U_a_*: 15, 10 and 5 keV (from left to right); Figure S2: In-plane M-H loops of ZCFO/PVDF-TrFE and BTO/ZCFO/PVDF-TrFE samples (a,c) in-plane and (b,d) out-of-plane for each sample, respectively; inset—the position of the sample in the magnetic field during the measurements; Figure S3: Representative AFM-mapping of ZCFO/PVDF-TrFE sample in morphology (top left), adhesion force (top right), mechanical stiffness (bottom left) and Young’s modulus (bottom right) contrast, obtained simultaneously.
