# Supplementary material for: Effect of Piezoelectric BaTiO3 Filler on Mechanical and Magnetoelectric Properties of Zn0.25Co0.75Fe2O4/PVDF-TrFE Composites
Source: Polymers (Basel). 2022 Nov 8;14(22):4807. doi: 10.3390/polym14224807 (PMC9695481; doi:10.3390/polym14224807)
Supplement: Supplementary file 1 [file polymers-14-04807-s001.zip › polymers-1996171-supplementary.pdf]

Supplementary materials

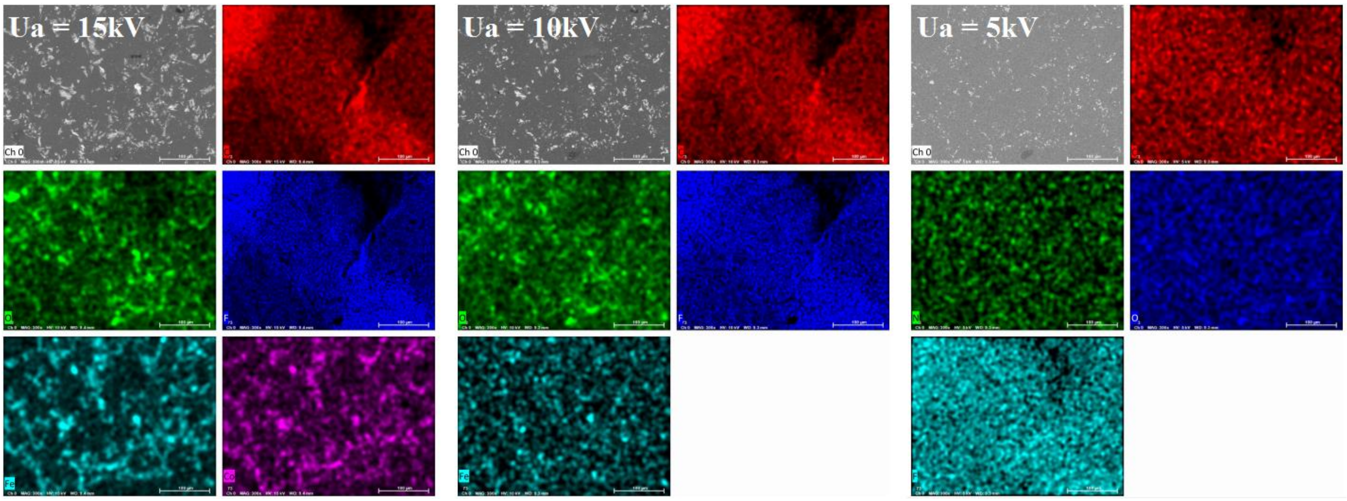

Figure S1. Representative EDX mapping, obtained for ZCFO/PVDF-TrFE sample, with different U_a: 15, 10 and 5 keV (from left to right).

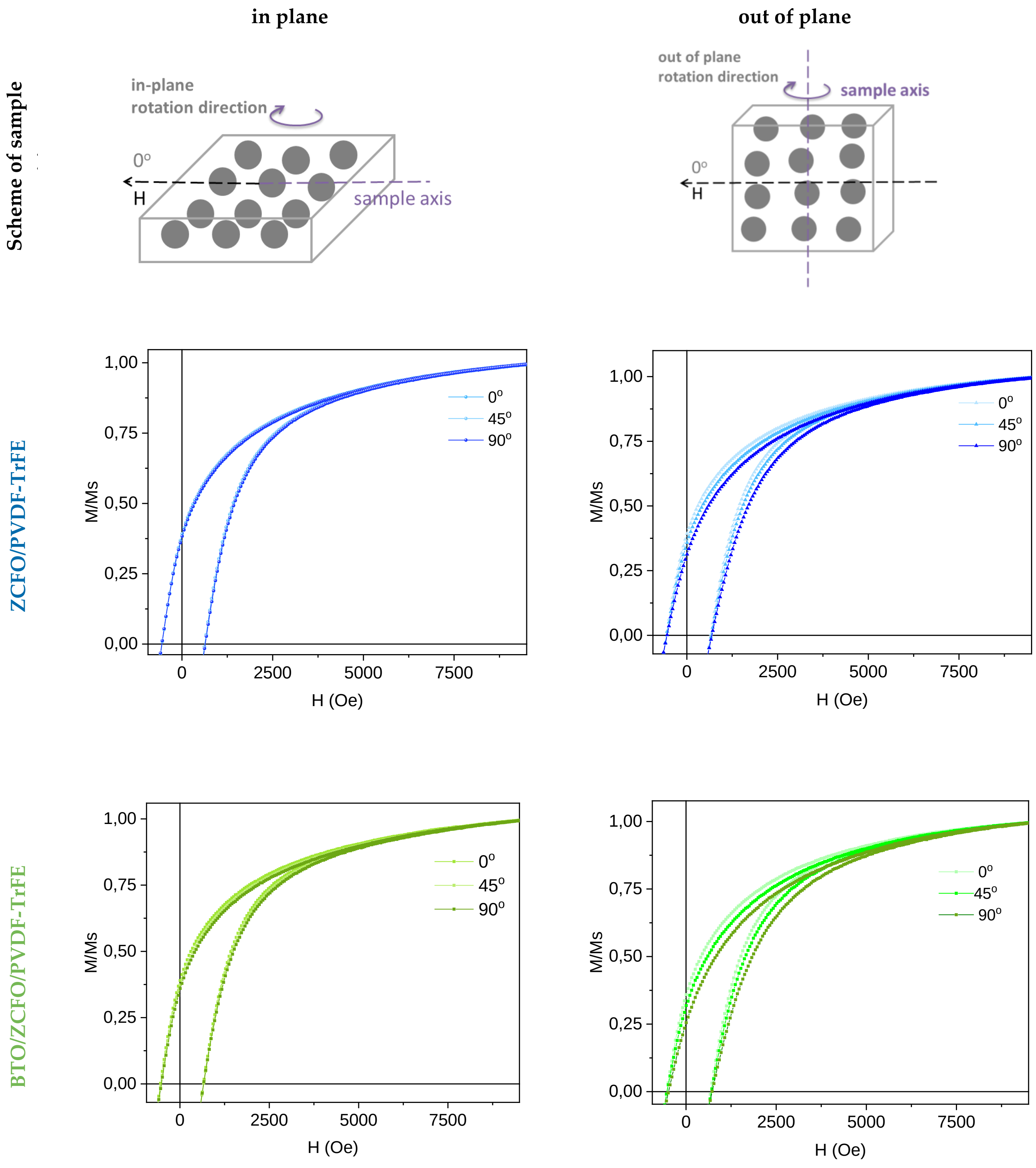

Figure S2. In-plane M-H loops of ZCFO/PVDF-TrFE and BTO/ZCFO/PVDF-TrFE samples (a,c) in-plane and (b,d) out-of-plane for each sample, respectively; inset – the position of the sample in the magnetic field during the measurements.

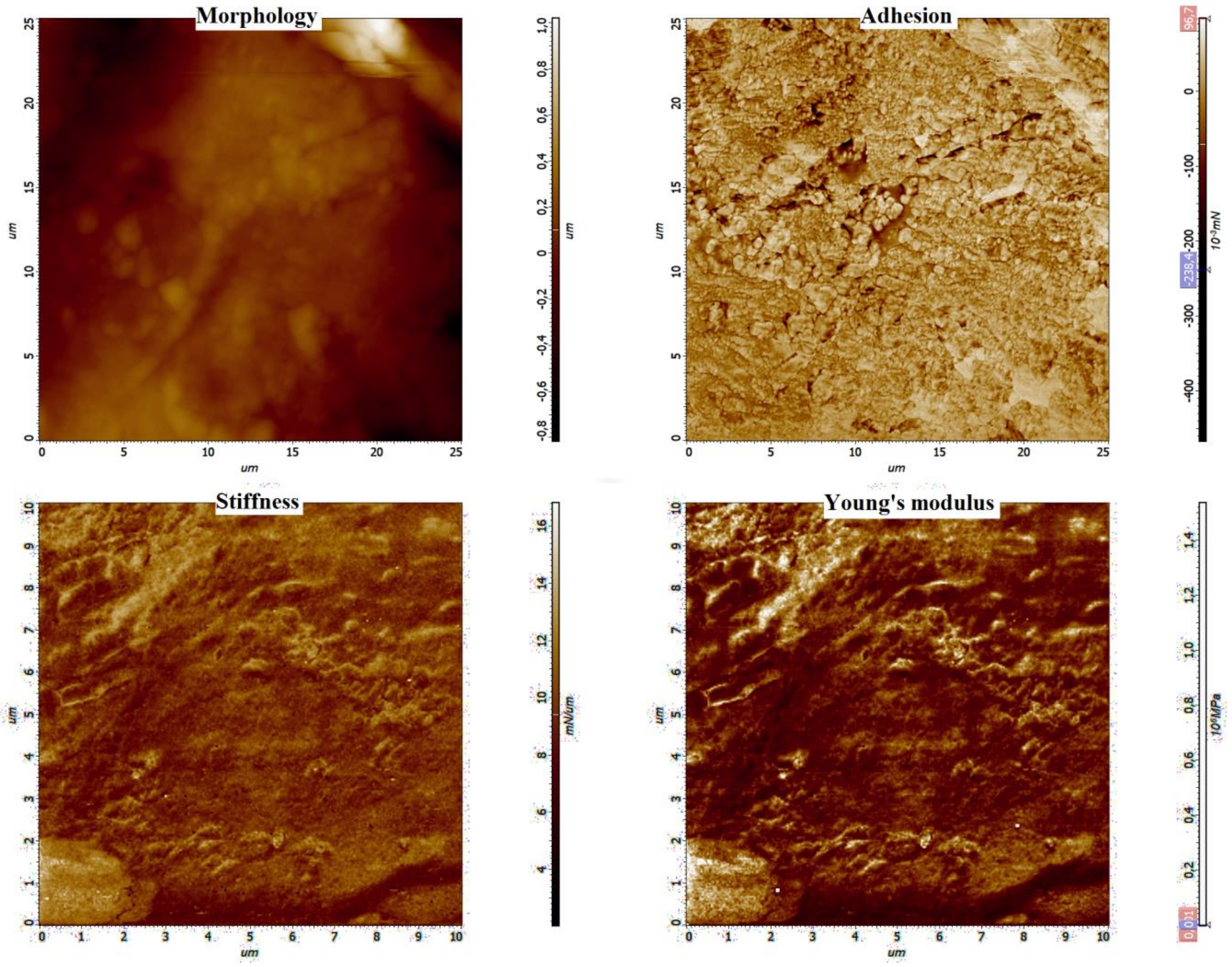

Figure S3. Representative AFM-mapping of ZCFO/PVDF-TrFE sample in morphology (top left), adhesion force (top right), mechanical stiffness (bottom left) and Young's modulus (bottom right) contrast, obtained simultaneously.
